# Delayed Onset Atrioventricular Block After Surgical Aortic Valve Replacement: A Rare Entity

**DOI:** 10.7759/cureus.25606

**Published:** 2022-06-02

**Authors:** Rajwinder Gill, Vineet Meghrajani, Shaharyar Ali, Maria Riasat

**Affiliations:** 1 Internal Medicine, Icahn School of Medicine at Mount Sinai Beth Israel, New York, USA; 2 Department of Cardiology, Icahn School of Medicine at Mount Sinai Beth Israel, New York, USA

**Keywords:** complete av block, savr, tavr, permanent pacemaker implantation (ppm), surgical aortic valve replacement (savr), av block

## Abstract

Surgical aortic valve replacement (SAVR) is the mainstay treatment for aortic valve diseases in patients with low surgical risk. Trans aortic valve replacement (TAVR) has also grown over the past few years, although limited durability data is available. Atrioventricular conduction abnormalities (AVCA) are known complications in the immediate period post-TAVR and SAVR. There are no case reports regarding the development of the delayed onset AVCA years after SAVR. In this case report, we present a male patient who developed a complete heart block six years after SAVR, following which he got the permanent pacemaker implantation (PPMI).

## Introduction

Surgical aortic valve replacement (SAVR) is a gold standard treatment for patients with aortic valve disease. In patients with a high prohibitive risk for SAVR, trans aortic valve replacement (TAVR) is a treatment option. In patients with both SAVR and TAVR as an option, shared decision-making needs to be done given the limited data available for TAVR. Atrioventricular conduction abnormalities (AVCA) have been described after SAVR and TAVR in the immediate postoperative period [1]. The delayed occurrence of AVCA after SAVR is infrequent. 

## Case presentation

A 56-year-old male with a history of hypertension, type 2 diabetes mellitus, coronary artery disease (CAD) status post (s/p)coronary artery bypass graft (CABG) in 2013, aortic insufficiency s/p mechanical valve replacement (MVR) in 2014, heart failure with reduced ejection fraction (HFrEF) presented with left-sided, intermittent, crushing chest pain from two days associated with exertion. He denied palpitations, nausea, vomiting, diaphoresis, leg swelling, abdominal pain, or fever/chills. 

**Figure 1:**
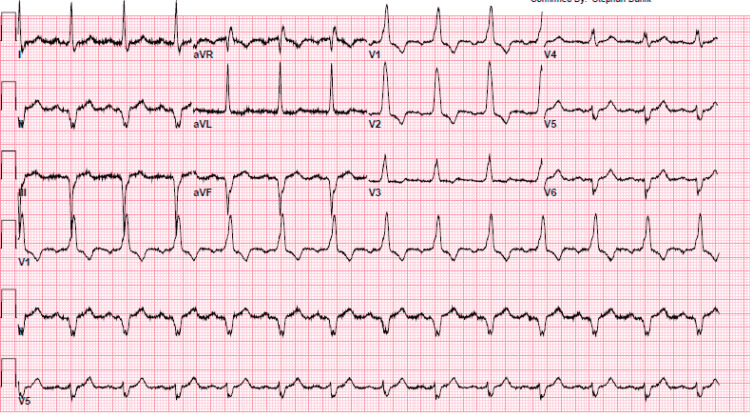
Old electrocardiogram (ECG) of the patient showing right bundle branch block (RBBB) done post-SAVR

**Figure 2:**
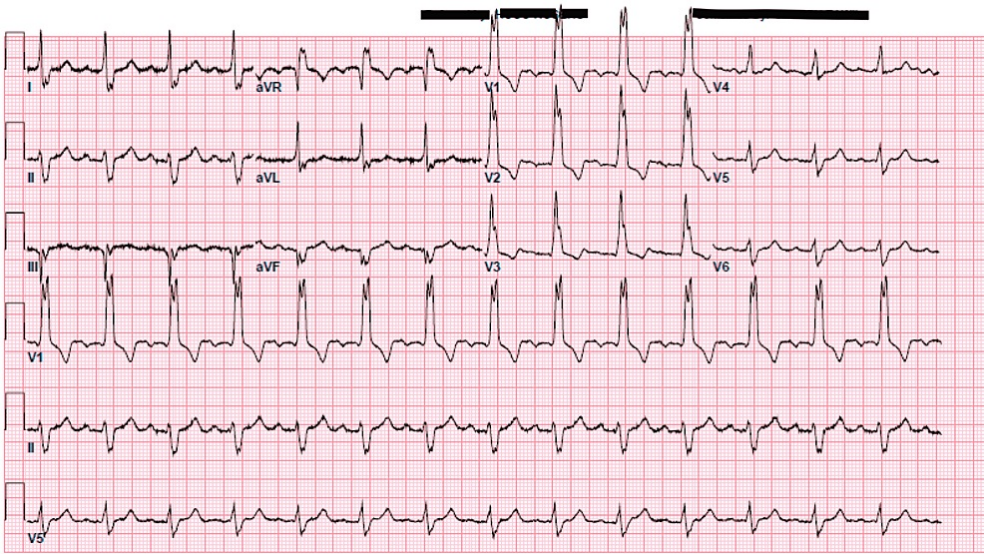
Initial electrocardiogram (ECG) showing right bundle branch block without any ST-T wave changes

Initial vital signs were noticeable a heart rate of 82, a temperature of 38 degrees Celsius, blood pressure of 128/76, a respiratory rate of 16 per minute, and oxygen saturation of 98% on room air. The cardiovascular exam was unremarkable. The initial electrocardiogram (ECG) was remarkable for the right bundle branch block (RBBB) without any ischemic changes, which were unchanged from the prior EKG (Figures 1-2). Troponin was normal in an initial set of labs. Left heart catheterization (LHC) was done via the right radial approach without any complications during the procedure, and it showed patent grafts. No intervention was done during the LHC. The patient didn't develop any hematoma post catheterization.

**Figure 3:**
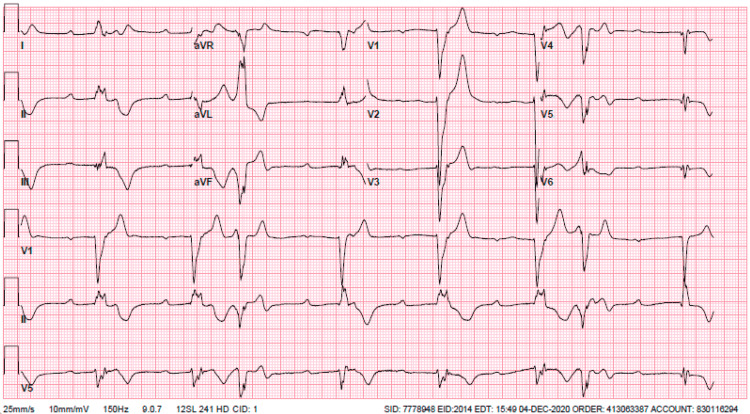
ECG showing complete heart block on the second day of hospitalization

**Figure 4:**
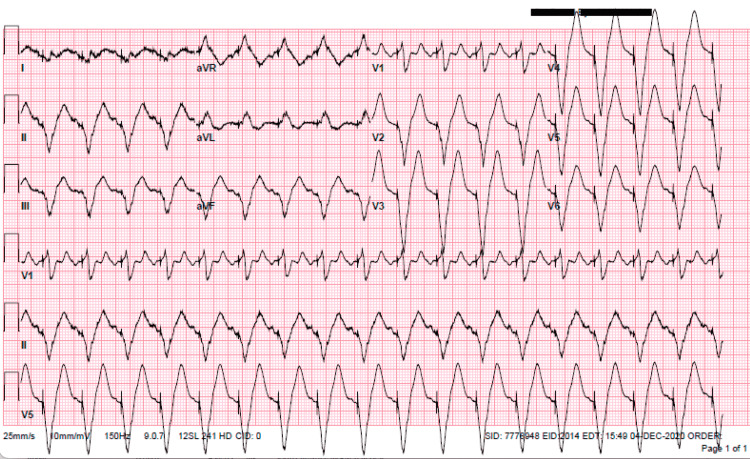
ECG after placement of biventricular pacemaker showing the atrial sensed ventricular paced rhythm

**Figure 5:**
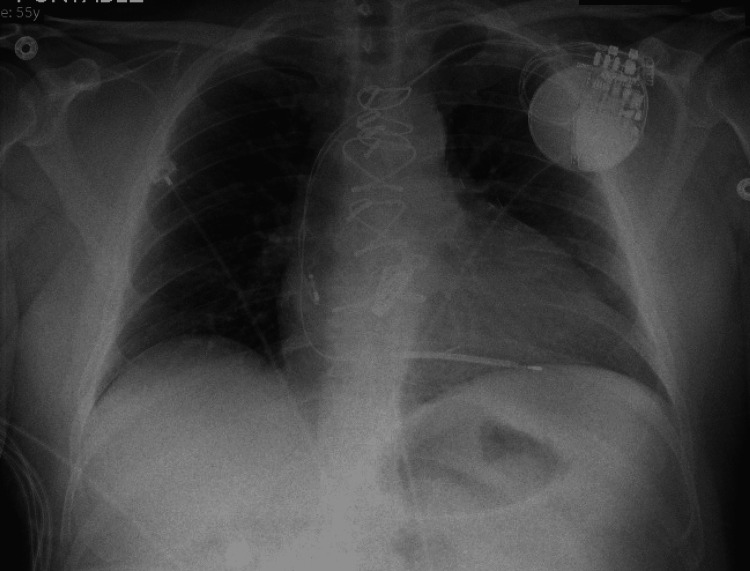
Chest X-ray after the placement of a pacemaker

The patient was kept in hospital to transition from heparin to warfarin. Three days after the LHC, he was found to be bradycardic to 30 on telemetry, and EKG was significant for ventricular escape rhythm and atrioventricular (AV) dissociation consistent with third-degree heart block (Figure 3). All the reversible causes of complete heart block were ruled out. Electrolytes and thyroid-stimulating hormone (TSH) were normal labs that day. The patient didn't have any signs of infection, and urinalysis, chest X-ray, and white blood cell count were normal. Laboratory workup on that day is shown in Table 1. The patient was on a beta-blocker which was held after the complete heart block. A transthoracic echocardiogram (TTE) showed severely reduced systolic function of the left ventricle with an ejection fraction (EF) of 27% (Video 1). All the native valves, including prosthetic, were normal on TTE. The patient didn't have any skin rash or any history of skin lesions suspicious of Lyme disease. The patient was asymptomatic and was transferred to the cardiac intensive care unit (ICU), and a biventricular pacemaker was placed without any complication during the procedure (Figures 4-5). He had three episodes of bradycardia before getting the pacemaker placed, and atropine was given during one of the episodes, following which bradycardia improved. Indication for cardiac resynchronization therapy (CRT-D) was QRS duration of >150 ms and EF of <35%.

**Table 1:** Laboratory work on the day of complete heart block pCO_2_ - partial pressure of carbon dioxide

Test	Results	Reference range
White blood count (WBC)	6.7	4.5-11.00 k/uL
Hemoglobin	15	13.6-16.3 G/DL
Platelet	306	150-450 k/uL
Sodium	138	135-145 meq/L
Potassium	4.8	3.5-5.2 mmol/L
Chloride	100	96-108 mmol/L
Phosphorus	2.7	2.4-4.7 mg/dL
Magnesium	2.1	1.5-2.5 mg/dL
Creatinine	0.91	0.5-1.1 MG/DL
Blood urea nitrogen	18	6-23 MG/DL
Brain natriuretic peptide (BNP)	89.5	0.0-100 pg/mL
Troponin	<0.010	<0.031 mg/dl
Aspartate aminotransferase	26	1-35 U/L
Alanine aminotransferase	28	1-45 U/L
Alkaline phosphatase	56	38-126 U/L
pH	7.37	7.35-7.45
pCO_2_	40	35-45 mmHg
Bicarbonate	26	21-29 mEq/L
Lactic acid	1.1	0.50-2.00 mmol/L
thyroid-stimulating hormone (TSH)	1.3869	0.4-4.2 uIU/mL

**Video 1:** Transthoracic echocardiogram (TTE) showing the dilated left ventricle and severely reduced left ventricular systolic function

## Discussion

SAVR and TAVR are the main treatment options for patients with symptomatic aortic valve disease. Due to the proximity of the infranodal conduction system to the aortic valvular apparatus, patients with aortic valve disorders frequently present with AVCA [1]. Complications such as AVCA, new left bundle branch block (LBBB), and various grades of intranodal AV block, including complete heart block after TAVR, are well described. AVCA most commonly occurs intraoperatively or during the immediate postoperative period. Very late cases of AV block after TAVR have been reported but are rare [2]. 

Rates of AVCA reported after SAVR are lower as compared to TAVR, and a permanent pacemaker (PPM) is implanted in only 3-4% of patients undergoing SAVR [1]. The study, comparing the patients' post-TAVR and SAVR with similar baseline abnormalities, found that 7.3% of the patients in TAVR had permanent pacemaker implantation (PPMI), a rate significantly higher than the rate of 3.4% observed in SAVR patients [3].

There is not a lot of literature on long-term follow-up of SAVR patients. Patients who underwent SAVR have a 2% incidence of pacemaker insertion within 30 days and 4% thereafter at a median follow-up of 3.76 years. There seems to be a persistent 1% annual risk for pacemaker insertion post-surgery in the first years after SAVR [4].

Up to 90% of PPMI is performed in the first week after TAVR, with the majority (97%) being done during the index hospitalization [5-6]. Sudden deaths in post-TAVR patients amount to 0.8-5.6% of all deaths and are often attributed to complete AV block [7].

MARE study (Ambulatory Electrocardiographic Monitoring for the Detection of High-Degree Atrio-Ventricular Block in Patients With New-onset PeRsistent LEft Bundle Branch Block After Transcatheter Aortic Valve Implantation) used the implantable cardiac monitor in LBBB post-TAVR recipients, which showed a high burden of arrhythmic events at one-year follow-up in close to 50% of the patients, leading to a treatment change in more than one-third of them. Significant bradyarrhythmias were detected in 20% of patients, with PPM required in nearly one-half of them [8].

Existing literature regarding predictors of AV block in patients going for AVR shows the most common indication for PPMI in post AVR patients is a complete atrioventricular block (AVB) followed by severe symptomatic bradycardia (SB). The presence of preexisting RBBB was a powerful risk factor for PPMI in the TAVR group but not in the SAVR group. No variable was found to be associated with PPMI after SAVR in the multivariate analysis in this study [3].

Most patients in both groups needed PPMI because complete AVB occurs within 24 hours after the procedure in most cases [4]. Some literature shows that RBBB is a predictor for early PPMI, atrial fibrillation (AF), and LBBB were predictors for late PPMI [9].

There is not enough data regarding the predictors of developing heart block post-SAVR, although there are few studies regarding TAVR. Follow-up of the patients getting the PPMI post-AVR is also conflicting, with one study showing the reduced EF on two-year follow-up and another study showing higher rates of hospitalization and mortality in the people getting PPMI [5-6].

In our patient, the cause of complete heart block was irreversible. Reversible causes of heart block were ruled out. The patient was on a beta-blocker which was stopped after spotting the complete heart block. There was no movement of the implanted valve. On the follow-up, he was pacemaker dependent. 

## Conclusions

Patients with SAVR can present with AV block years after their surgery. Our patient developed a complete heart block six years after surgery leading to PPMI. The pathophysiology of developing delayed-onset complete heart block is not clear. It may be related to fibrosis or micromovement of the valve. It is extremely important to study more regarding the delayed onset AVCA requiring PPMI as it can lead to sudden cardiac death. Patients post SAVR are at high risk of developing AVCA at any point in life and should be monitored closely by cardiology. 
